# Effective treatment of aquaculture wastewater with mussel/microalgae/bacteria complex ecosystem: a pilot study

**DOI:** 10.1038/s41598-021-04499-8

**Published:** 2022-02-10

**Authors:** Bing Geng, Yongchao Li, Xue Liu, Jing Ye, Weifeng Guo

**Affiliations:** 1 Institute of Environment and Sustainable Development in Agriculture, Chinese Academy of Agricultural Sciences, Beijing, 100081 China; 2 Key Laboratory of Recycling and Eco-Treatment of Waste Biomass of Zhejiang Province, Zhejiang University of Science and Technology, Hangzhou, 310023 China; 3 Zhejiang Qinghu Agricultural Science and Technology Co. Ltd, Shaoxing, 312000 China

**Keywords:** Environmental chemistry, Bioremediation, Environmental microbiology

## Abstract

The discharge of aquaculture wastewater increased significantly in China. Especially, high content of nitrogen and phosphorus in wastewater could destroy the receiving water environment. To reduce the pollution of aquaculture wastewater, farmed triangle sail mussel (*Hyriopsis cumingii*) was proposed to be cultivated in the river. This was the first time that bacteria (*Bacillus subtilis* and *Bacillus licheniformis*) and microalgae (*Chlorella vulgaris*) were also used and complemented ecosystem functions. The pollutants in wastewater were assimilated by *Chlorella vulgaris* biomass*,* which was then removed through continuous filter-feeding of *Hyriopsis cumingii*. While, *Bacillus subtilis* and *Bacillus licheniformis* enhanced the digestive enzyme activities of mussel. It demonstrated that approximately 4 mussels/m^3^ was the optimal breeding density. Under such condition, orthogonal experiment indicated that the dose of *Bacillus subtilis*, *Bacillus licheniformis*, and *Chlorella vulgaris* should be 0.5, 1, and 2 mL respectively. Compared with mussel, mussel/microalgae, mussel/bacteria system, treatment ability of the mussel/microalgae/bacteria system in batch experiment was better, and 94.67% of NH_3_-N, 92.89% of TP and 77.78% of COD were reduced after reaction for 6 days. Finally, 90 thousand mussels per hectare of water were cultivated in Kulv river in China, and the field experiment showed that water quality was significantly improved. After about 35 days of operation, NH_3_-N, TN, TP and COD concentration were maintained around 0.3, 0.8, 0.3, and 30 mg/L respectively. Therefore, the mussel/microalgae /bacteria system in this study showed a sustainable and efficient characteristic of aquaculture wastewater bioremediation.

## Introduction

In order to satisfy the increasing consumption of aquatic product, aquaculture industry in China has been intensely developed in recent decades^1,2^. However, large amount of effluent from aquaculture industry was also generated without proper treatment, and caused severe problems to nearby water environment. More importantly, the aquaculture wastewater was usually rich in nutrients (nitrogen, phosphorus) and dissolved organic carbon, which could potentially cause harmful algal blooms and damage the natural aquatic ecosystem^3,4^. Up to now, many different kinds of techniques have been used to treat aquaculture wastewater. For example, nitrogenous compounds in water can be transformed into gaseous N_2_ by biological nitrification/denitrification processes. Phosphorus can be removed through phosphorus-accumulating bacteria or chemical precipitation^5,6^. These techniques were commonly adopted in sewage treatment plant. However, it was still challenging to treat river water which was polluted by aquaculture wastewater.

Several studies showed that filter-feeding animals such as planktivorous mussel could improve water quality of aquaculture ponds, because with the growth of mussel they consumed phytoplankton, suspended solid, and alternated nutrient cycles. Nowadays, mussel farming has been thrived in some countries, since they can also be used as food through the growth and harvesting of mussels^7–10^. Specially, triangle sail mussel *Hyriopsis cumingii* was one of the most important Chinese endemic mussel species, which was widely used for freshwater pearl production^11^. It was also exploited as a biomanipulation tool for suppression of cyanobacterial bloom in eutrophic shallow lakes and ponds^12,13^. Moreover, mussel cultivation can be operated at a lower cost compared with some land-based methods which was used to improve coastal water quality^8,14,15^. However, one drawback of mussel farming for water treatment was the uncertainty associated with actual operating process. It may even deteriorate water quality due to excreted feces from the farmed mussels at the site, which made a leakage of ammonia and phosphorus to river water^16^. However, there is no study about the efficiency improvement and breeding methods of mussel farming when they are used as a nutrient-cleaning option.

Microalgae can grow rapidly in aquaculture wastewater. Meanwhile, it can accumulate protein or lipid, which can be subsequently used to produce aquaculture feed and biofuel^17,18^. Moreover, it can remove nitrogen and phosphorus from wastewater at low operational costs^19,20^. Ammonium and nitrate can not only be taken up by microalgae to synthesize biomass, but also eliminated by algae-induced nitrification and denitrification process. P can be removed through a combination of adsorption, algae-induced chemical precipitation, and microbial reactions^21,22^. In this process, no toxic substances were produced, and microalgae biomass can also be harvested and then processed into products with high-value. Nonetheless, traditional algae-based water treatment systems encountered some problems, such as low biomass of microalgae, and difficult harvesting^23^.

Furthermore, *Bacillus* species were good candidates for bio-remediation^24,25^. It was documented that with addition of 10^8^ CFU/mL *Bacillus subtilis* strain directly to rearing water, nitrite, ammonia, and nitrate concentration was significantly reduced to a tolerable range for shrimp aquaculture^26^. Due to production of extracellular alkaline keratinase and disulfide reductase, *Bacillus* were also useful in decomposing macromolecular organic waste in water, which came from feces, remains, and suspended solids^27,28^. Additionally, some types of *Bacillus* had good probiotic effect via producing antimicrobial peptides to inhibit certain pathogenic bacteria of fish^29^.

Above all, mussel cultivated in the river can remove pollutants. Despite the well-known advantages of microalgae and *Bacillus* species, they were seldom used to improve remediation efficiency of traditional mussel farming. Aiming at the problem of water pollution in high-density aquaculture ponds, a study was carried out to restore aquaculture wastewater with the combined ecosystem of mussels and microalgae/bacteria. The main objectives were to: (1) study the characteristic of mussel and examine its breeding density; (2) consider the effect of bacteria and microalgae dose on pollutant removal ability; (3) investigate wastewater treatment performance and digestive enzyme activities in mussels, mussel/microalgae, mussels/bacteria, and mussel/microalgae/bacteria systems respectively, (4) design and conduct continuous treatment of real pond water. Finally, a low-cost and highly efficient technique for treating the aquaculture wastewater was obtained.

## Materials and methods

### Materials

**Figure 1:**
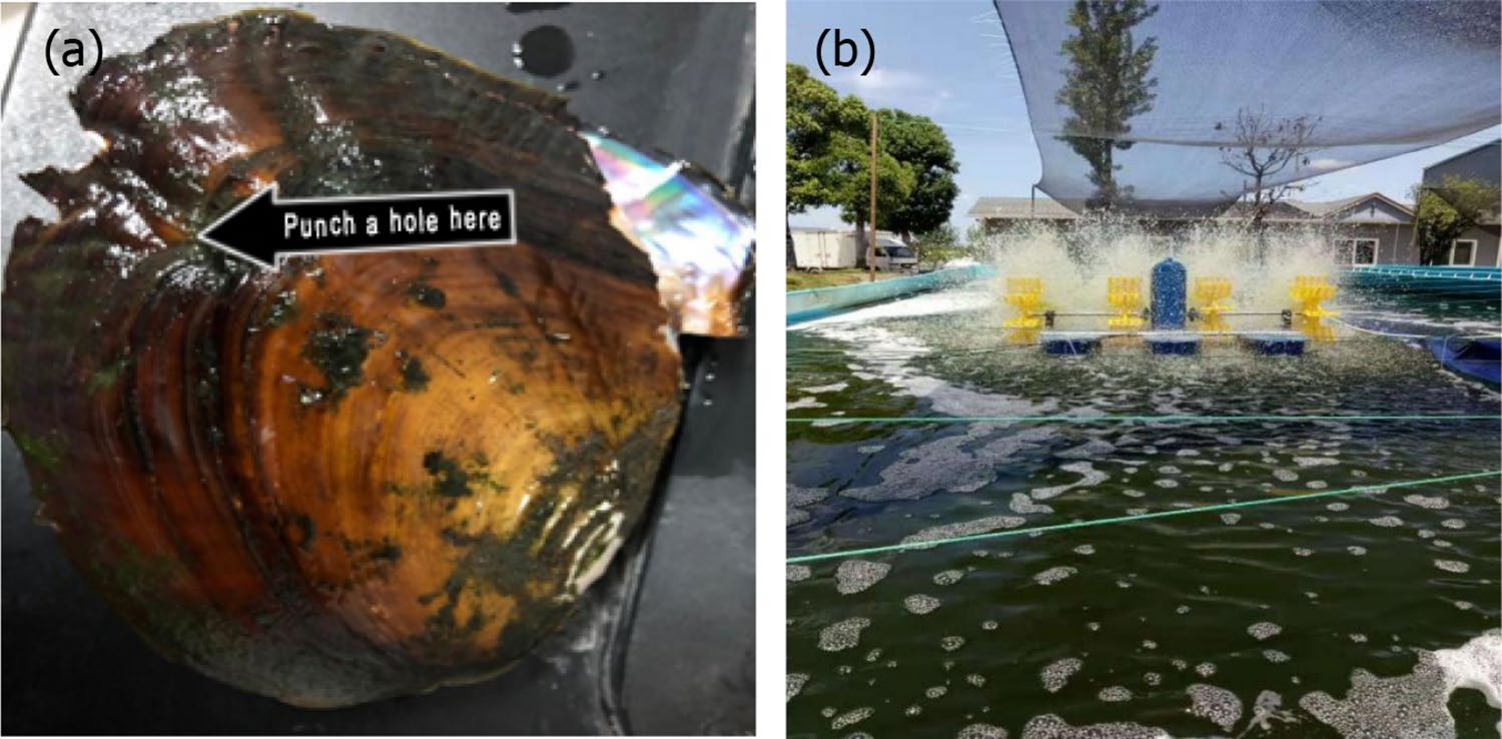
(**a**) Adult mussel used in the study, (**b**) large-scale cultivation of microalgae in a tank.

The adult mussels (*Hyriopsis cumingii*) were provided by Zhejiang Qinghu Agricultural Science and Technology Co., Ltd. As shown in Fig. 1a, mussels (400 ± 50 g in wet weight, 14 ± 2.2 cm in shell length, 9 ± 0.5 cm in shell width, 4.3 ± 0.2 cm in shell height) with strong water spray were selected. In order to reduce the external influence on the wastewater treatment experiment, the selected mussels were placed into tap water for 2–3 day after impurities on shell surface was cleaned first.

*Chlorella vulgaris* which had high photosynthetic efficiency, short breeding cycle, and strong environmental adaptability was chosen in this study. They were obtained from the Institute of Hydrobiology at the Chinese Academy of Sciences, and precultured in bottles containing liquid BG-11 medium. To maintain their growth, *Chlorella vulgaris* were put in an illumination incubator with 12:12 light: dark cycles. The microalgae cells reached exponential phase on day 7. As shown in Fig. 1b, large-scale *Chlorella vulgaris* were cultivated in a tank where microalgae biomass concentration was about 10 mg/L.

*Bacillus subtilis* strain YFFJ-2 and *Bacillus licheniformis* strain NJ-6 were isolated and screened in our laboratory. Their physiological and biochemical characteristics are shown in Table 1. *Bacillus subtilis* strain YFFJ-2 were cultivated in sealed glass bottles which contained the following components: 4.0 g/L beef extract, 3.8 g/L peptone, 3.3 g/L NaCl, 4.0 g/L glucose, 3.3 g/L starch, 3.4 g/L KH_2_PO_4_, 0.2 g/L MnSO_4_·7H_2_O. After 16 h of cultivation, the bacteria of exponential phase were harvested and used in the following experiment. *Bacillus licheniformis* strain NJ-6 were cultivated in medium which contained 2.0 g/L beef extract, 5.0 g/L peptone, 3.0 g/L yeast powder, 0.005 g/L MnSO_4_, 2.0 g/L NaCl, 3.0 g/L K_2_HPO_4_, 0.02 g/L MgSO_4_. The biomass was harvested after 48 h of cultivation.

**Table 1:** Physiological and biochemical characteristics of *Bacillus* strain YFFJ-2, NJ-6 strain.

Characteristics	YFFJ-2	NJ-6
Source	Duck-manure	Compost of excrement
Gram staining	+	+
Respiration type	Aerobic	Facultative aerobic
Morphology	Rod shape	Rod or fusiform shape
Spore location	In the end or middle	In the end
Temperature (°C)	15 ~ 55	15 ~ 55
pH	4.5 ~ 10.0	4.5 ~ 10.0
Catalase	+	+
Voges-Proskauer test	+	+
Nitrate reductase	+	+
H_2_S production	−	−
Indole test	−	−
Glucose	+	+
Raffinose	+	+
Arabinose	+	+
Sucrose	+	+
Mannitol	+	+
Xylose	+	+
Maltose	+	+
Fructose	+	+
Lactose	+	+
Hydrolysis of starch	+	+
Hydrolysis of gelatin	+	+

+ positive reaction; −negative reaction.

### Investigation of mussel breeding density

The influence of mussel breeding density on wastewater treatment performance was investigated. Aquaculture wastewater was collected from a fish-farming area in Hefei city of China. The concentration of total nitrogen (TN), total phosphorus (TP), suspended solid (SS), ammonia nitrogen (NH_3_-N) and dissolved oxygen (DO) in tested wastewater samples was 3.0, 0.3, 95.0, 2.0, 6.2 mg/L, respectively. The experiments were conducted in several rectangular plastic tanks (2 m in length, 0.5 m in width). 5 cm-thick sediment from fish farming zone was first laid on the bottom of tanks, and 0, 2, 4, and 8 mussels were put in each tank containing 1000 L aquaculture wastewater, separately. Batch experiment was then conducted in outdoor under the natural environment. 50 mL of water sample was taken from each tank per day, and TP and NH_3_-N concentration were analyzed. At the same time, pH and DO were recorded during the whole process.

### Influence of bacteria and microalgae dose on the water quality

After the optional density of mussel was settled, an orthogonal experiment was carried out to explore the effect of bacteria and microalgae dose on wastewater treatment efficiency. Our previous experiments suggested that when the microalgae amount was more than 3 mL, filter-feeding ability of mussel would be inhibited by rapid growth of microalgae, resulting in increase of turbidity and chromaticity in water. So, the volume of microalgae was set around 1–3 mL. When too much *Bacillus subtilis* and *Bacillus licheniformis* biomass was added to wastewater, their massive reproduction could consume most of oxygen in water, leading to significant decrease of DO concentration which was bad for the growth of mussels. So, 0.5–2 mL was chosen as the dose for bacteria. Thus, inoculation quantity of bacteria and microalgae on NH_3_-N removal ability was investigated in this study. Three levels parameters of orthogonal experimental are shown in Table 2.

**Table 2:** The level and value range of different factors in orthogonal experimental parameters.

Test number	A (mL)	B (mL)	C (mL)
	*Bacillus subtilis*	*Bacillus licheniformis*	*Chlorella vulgaris*
1	0.5	0.5	1.0
2	1.0	1.0	2.0
3	2.0	2.0	3.0

### Wastewater treatment by mussel/microalgae/bacteria in batch experiment

Batch experiment was undertaken to investigate pollutant removal ability by mussels in the presence of bacteria and microalgae, which was named as mussel/microalgae/bacteria ecosystem. The procedure was described as follows. First, several mussels (*Hyriopsis cumingii*) were put in rectangular plastic tanks containing 1000 L aquaculture wastewater, then certain volume of bacteria (*Bacillus subtilis* YFFJ-2 and *Bacillus licheniformis* NJ-6) and microalgae (*Chlorella vulgaris*) with optimal inoculation quantity were added. The whole experiment was conducted in outdoor under natural environment for 6 days. 20 mL of supernate was taken from the tank at 12:00 PM every day, and residual COD, TP and NH_3_-N in wastewater were analyzed. In addition, aquaculture wastewater treatment by *Hyriopsis cumingii* alone (named as mussel system) was conducted as described above. Aquaculture wastewater treatment performance by *Hyriopsis cumingii* with adding *Bacillus subtilis* YFFJ-2 and *Bacillus licheniformis* NJ-6 (named as mussel/bacteria system) was also studied. Moreover, COD, TP and NH_3_-N removal ability by *Hyriopsis cumingii* along with *Chlorella vulgaris* (named as mussel/algae system) was investigated.

After the treatment, three mussels were sampled from each system for measuring the activities of digestive enzymes in their digestive gland and stomach. First, these mussels were opened by a special shell opener, and stomachs were cut out without damage. Then, they were flushed with ice-cold sterilized saline water to remove body fluids and some impurities, and excess saline water was wiped out with filter paper. About 0.3 g of digestive gland tissue samples were centrifuged (12,000 rpm) for 10 min at 4 °C, and the supernatant containing crude enzyme extract was obtained and frozen at − 80 °C. Amylase and pepsin activity were further studied using standard kits according to the manufacturer's instructions (Jiancheng Bioengineering Institute, Nanjing, China), respectively. The digestive enzyme activities were expressed in standard units (U/mg prot).

### Field experiment

**Figure 2:**
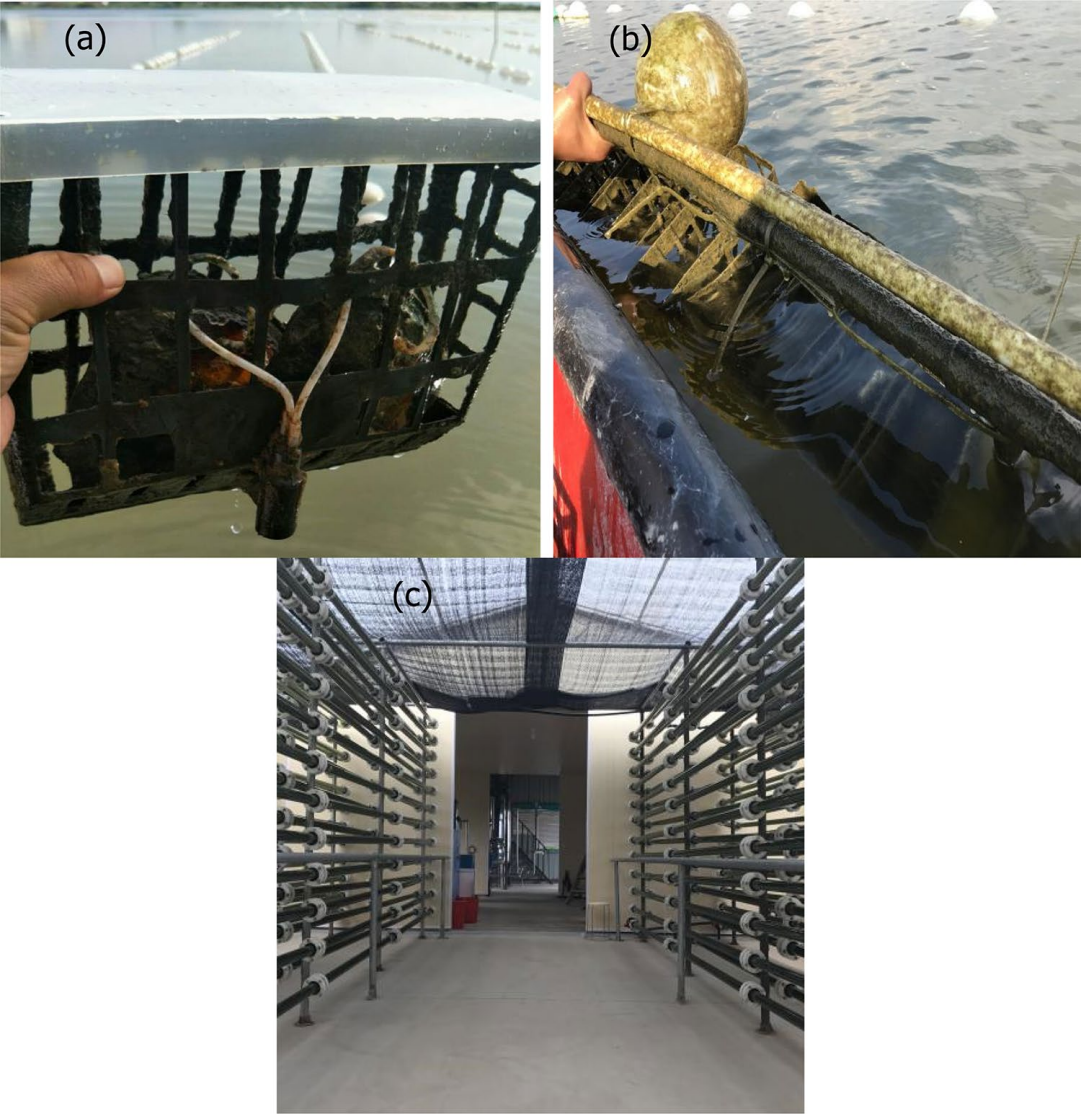
(**a**) Photos of foster boxes designed for mussels’ cultivation, (**b**) automatic jacking system connected with foster box, (**c**) pipe network of feeding for mussels.

Chaohu lake was one of five major lakes in the middle and lower reaches of Yangtze River. There were 35 rivers along the lake, and our study site was Kulv river (2–3 m deep) which was located in Feixi city. The increase of pond-farming and overuse of fertilizers at the upper reaches of Kulv river resulted in nitrogen and phosphorus pollution. Conventional aquaculture model was made of cylindrical nets in which the mussels were placed and then suspended for culture^30^. Different from this model, an automation pipe network supply feeding system was built in our study. It mainly included central controller, compounding tank, lifting pump, agitation equipment, feeding pipe network and multiple foster boxes (Fig. S1). The central controller was connected with compounding tank, lifting pump and mixing feed device, respectively. Mussels were placed inside the foster boxes (Fig. 2a) that were specifically designed to secure and maintain shellfish for aquaculture grow-out. The foster boxes were 25 m long, 0.15 m wide, and 0.3 m high. They were often set 0.5 m under the water surface through automatic jacking system (Fig. 2b). Under the management of central controller, bacteria solution was added into large-scale microalgae in the compounding tank, and then the formed mixture was transported to the mixing feed station through lifting pump. After mixing the microalgae and bacteria, the prepared ingredients were transported into pipe network. While, the small pipe in the foster boxes was connected with pipe network through a drilled hole on mussel (as shown in Fig. 1a). So, the formed nutrient mixture was directly fed to *hyriopsis cumingii* in the foster boxes through this automatic pipe network regularly (Fig. 2c), which can also prevent the discharge of nutrient into water. There were about 90 thousand mussels per hectare of water surface, and 120 thousand mussels were farmed in total. The filtering capacity of one mature mussel was about 50 L/day. So, mussels in one hectare could treat about 4500 m^3^ of aquaculture wastewater every day. The field experiment had run for several months. NH_3_-N, TN, COD and TP concentration of water samples were monitored every 7 days according to standard methods^31^. pH and DO were detected at 0.5 m below the water surface using a portable Hach (HQ40D) portable multi meter.

### Statistical analysis

All the batch and field experiments were carried out in triplicate, and collected data was expressed as mean ± SD. One-way ANOVA was performed with SPSS 17.0 software to determine the significant difference of digestive enzyme activities between different treatment systems. P < 0.05 was set as a significant difference.

### Ethical approval

This manuscript is an original work and never has been published elsewhere in any form or language.

## Results

### Influence of breeding density of mussels on water quality

It was known that mussel farming in the surrounding of beaches or semi-enclosed ponds can improve water transparency. However, high breeding density may cause negative impacts on the environment and increase the risk of hypoxia^32^. In our approach, the effect of mussel cultivation density on the changes of DO, TN, TP, pH value of aquaculture wastewater was studied.

**Figure 3:**
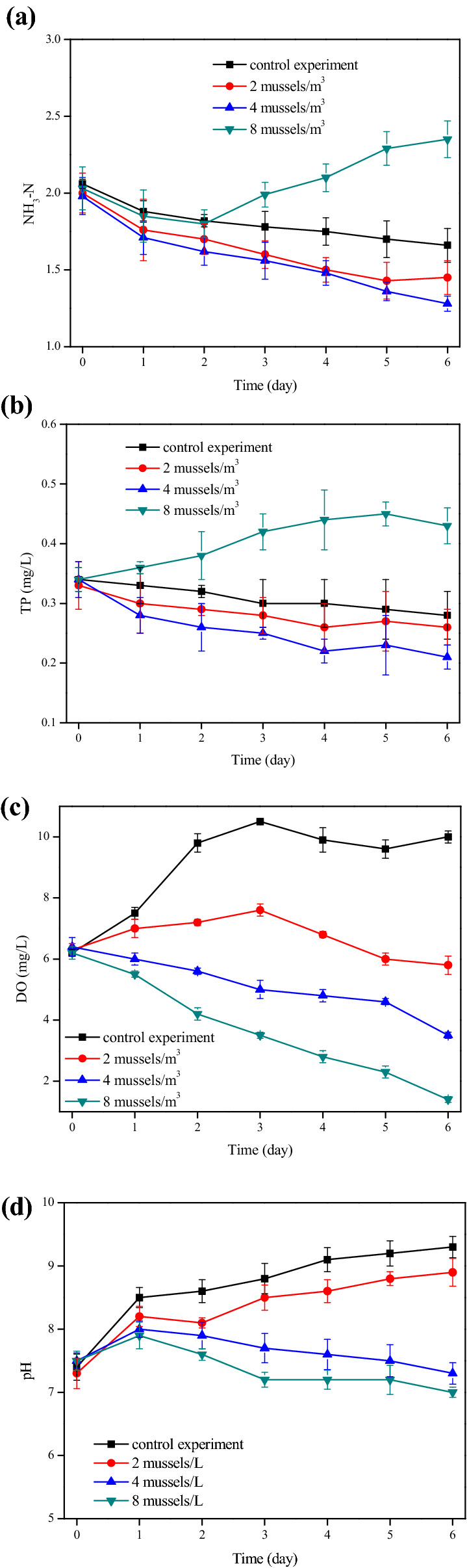
The variation of (**a**) NH_3_-N, (**b**) TP, (**c**) DO, and (**d**) pH concentration in mussel system with different breeding density.

As shown in Fig. 3a, when no mussel was added as the control, NH_3_-N content in the aquaculture wastewater was decreasing day by day. This was because there were already some microalgae in the original wastewater, which can digest inorganic nitrogen sources during their growth. Compared with the nitrate and nitrite, microalgae preferred ammonium since it can be assimilated by consuming less energy^33^. When 2 and 4 mussels/m^3^ was added, NH_3_-N concentration decreased distinctively, and was 1.45 and 1.48 mg/L on the 6th day, respectively. However, as the mussel density increased to 8 mussels/m^3^, the finial NH_3_-N concentration in water was greater than that in the raw wastewater. This indicated that certain density of mussels indeed removed partial nitrogen from the wastewater, however, when there were too many mussels, more nitrogen from their excrement was discharged to the water samples. As shown in Fig. 3b, when 2 or 4 mussels/m^3^ was cultivated, TP concentration in the water was less than that in the control experiment. Whereas, as the breeding density reached to 8 mussels/m^3^, the TP concentration increased significantly at the end of experiment. It was also found that the change of residual TP presented same tendency as that of NH_3_-N. This once again proved that high-density mussels were not suitable to the treatment of aquaculture wastewater.

DO and pH value were two very important indexes of water quality, and they were analyzed under different systems. As we can see from Fig. 3 c and d, in the control experiment the DO increased from initial 6.2 mg/L to about 10 mg/L on the 6th day, while pH increased to above 9.0. The important reason was that microalgae cultivated in aquaculture wastewater used solar energy to perform photosynthesis and synthesized carbohydrate from carbon dioxide, resulted in production of O_2_ and increase of pH value in water^34,35^. When mussels were added to aquaculture wastewater, the variation trend of DO concentration changed. Especially, when the density reached to 8 mussels/m^3^, DO value decreased seriously to 1.4 mg/L on the 6th day. However, environmental quality standards for surface water in China (GB 3838-2002) demanded that DO concentration in the surface water should be higher than 2.0 mg/L under normal circumstances^36^. Figure 3d showed that the pH value during the whole treatment process was in the range of 6–9. Furthermore, when mussels were cultivated in aquaculture wastewater, solution pH was lower than that in the control experiment.

### Optimization of bacteria and microalgae dose

As we know, *Bacillus subtilis*, *Bacillus licheniformis* and *Chlorella vulgaris* were important to bio-remediation of aquaculture wastewater. Under certain environmental condition, a balance can be achieved over time by mixing bacteria and microalgae, but there should be an optimized proportion of their doses which was good for ecosystem's functions. Table 3 shows the influence of *Bacillus subtilis* (A), *Bacillus licheniformis* (B) and *Chlorella vulgaris* (C) dose on wastewater treatment efficiency using an orthogonal test^37^. NH_3_-N removal was chosen as the main factor to decide the optimal ratio. When the factor was A, the level was 1 (0.5 mL), and the evaluation index was NH_3_-N removal efficiency, K_1_ was mathematically expressed by: K_1_ = (74.7% + 77.4% + 69.3%)/3 = 73.8%, which represented the average value of NH_3_-N removal efficiency for factor A at level 1. In this case, the value of K should be as large as possible. The optimal level of algae/bacteria amount was obtained by contrasting different K values. R indicated the significance of different levels on NH_3_-N removal, and the maximum value of R for certain factor implied it was the most important factor^38^.

**Table 3:** Analysis and results of orthogonal experimental design for bacteria/microalgae ratio.

Experiment	Factors (mL)			NH_3_-N removal
	A	B	C	
1	0.5	0.5	1.0	74.7%
2	0.5	1.0	2.0	77.4%
3	0.5	2.0	3.0	69.3%
4	1.0	0.5	2.0	76.5%
5	1.0	1.0	3.0	73.8%
6	1.0	2.0	1.0	70.5%
7	2.0	0.5	3.0	68.6%
8	2.0	1.0	1.0	72%
9	2.0	2.0	2.0	64.8%
**Removal efficiency**				
$\overline{{{\text{K}}_{1} }}$	73.8%	73.2%	72.4%	
$\overline{{{\text{K}}_{2} }}$	73.6%	74.4%	72.9%	
$\overline{{{\text{K}}_{3} }}$	68.5%	68.2%	70.6%	
R	5.3	6.2	2.3	
Order of importance	B>A>C			
Optimal level	A1	B2	C2	

### Treatment of aquaculture wastewater by mussels in different system

**Figure 4:**
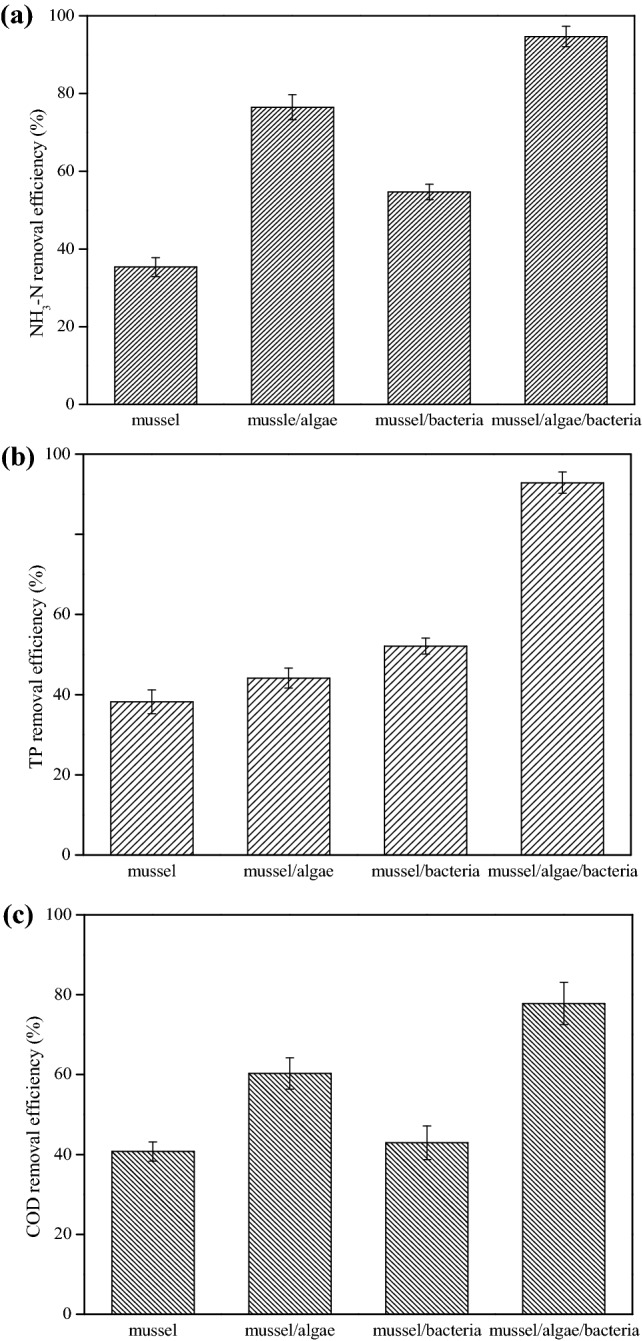
(**a**) NH_3_-N, (**b**) TP, and (**c**) COD removal ability in four different system.

Mussels with optimal density were cultivated in the batch experiment, while microalgae and bacteria with the optimal dose was added to the complex ecosystem. The change NH_3_-N, TP and COD concentration in four systems were studied at the same reaction condition. As shown in Fig. 4a, after 6 days of reaction, ammonia nitrogen removal efficiency in mussel, mussel/microalgae, mussel/bacteria, and mussel/microalgae/bacteria system was 35.4%, 76.5%, 54.7%, and 94.7%, respectively. It was obvious that with the simultaneous presence of *Bacillus subtilis*, *Bacillus licheniformis* and *Chlorella vulgaris*, NH_3_-N removal ability of *Hyriopsis cumingii* was significant enhanced. The result in Fig. 4b also implied that mussel/microalgae/bacteria complex system had better remediation ability towards nutrient P than the other systems. Although the COD removal ability in mussel/microalgae/bacteria ecosystem was not as high as that of the nutrient N and P, 77.8% of COD was still reduced finally. So, *Hyriopsis cumingii* farming with addition of *Bacillus subtilis*, *Bacillus licheniformis* and *Chlorella vulgaris* biomass was proposed as a tool to improve the quality of aquaculture wastewater in a field test.

### The changes of water quality in Kulv river installed with mussel/microalgae/bacteria culture

**Figure 5:**
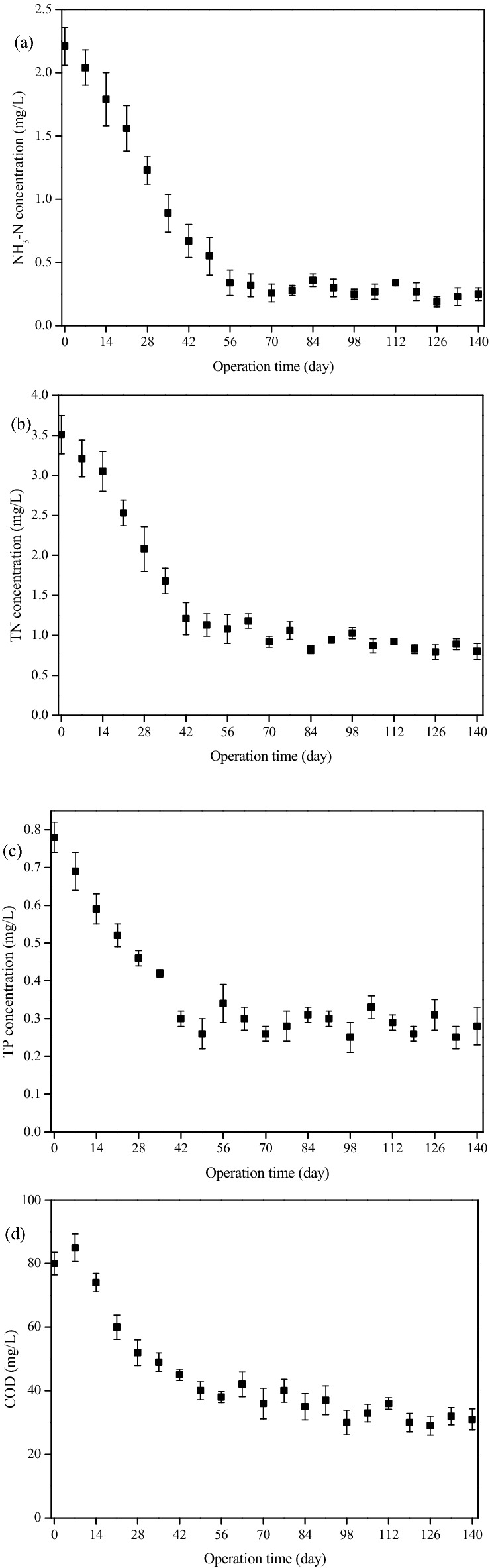
The change of (**a**) NH_3_-N, (**b**) TN, (**c**) TP, and (**d**) COD concentration in Kulv river installed with mussel/microalgae/bacteria treatment system.

The mussel/microalgae/bacteria treatment system began operation in May 2019, and the water quality was analyzed every 7 days. As shown in Fig. 5a, NH_3_-N concentration decreased from initial 2.2 mg/L to 0.89 mg/L on the 35th day, and then hovered around 0.3 mg/L. From Fig. 5 b, c, and d, it can also be seen that the change of TN, TP, COD concentration was similar to that of NH_3_-N. During the first 35 days, TN, TP, COD concentration dropped significantly, then maintained at about 0.8, 0.3, and 30 mg/L in the next 3 months, respectively. It indicated that the mussel/microalgae/bacteria treatment system began to work quickly, and water quality in Kulv river was gradually getting better despite the occasional discharge of wastewater in upstream. During the whole process, pH and DO concentration of the river water was in the range of 6.5–7.5 and 5.0–5.5 mg/L, separately. More importantly, after the mussel/microalgae/bacteria system run for 42 days, pH, NH_3_-N, TN, COD and TP concentration in Kulv river reached the required standard of Class IV in environmental quality standards for surface water in China (GB 3838–2002)^36^.

## Discussion

Optimal composition in mussel/microalgae/bacteria system was first discussed in our study. The effect of breeding density of mussels on water quality demonstrated that although the mussels can effectively remove nitrogen and phosphorus from aquaculture wastewater, high density of mussels (8 mussels/m^3^) led to the degradation of water quality. Plus, the triangle sail mussel *Hyriopsis cumingii* species was commonly cultivated for commercial freshwater pearl production in China^11^. It was expected that the mussel farming would not only achieve good effect of water treatment but also meet economic benefit. So, higher breeding density for mussel was encouraged, and approximately 4 mussels/m^3^ was recommended as the optimal breeding density of mussels in this study. As shown in Table 3, the dose of bacteria and microalgae influenced pollutant removal performance. Specially, *Bacillus licheniformis* dose performed an important role in NH_3_-N removal ability, followed by *Bacillus subtilis* and *Chlorella vulgaris* amount. The compositional parameters for obtaining an optimal NH_3_-N removal efficiency were A1 (0.5 mL), B2 (1 mL), and C2 (2 mL).

The optimized technology for pearl mussel farming in pearl culture with the presence of Chinese bighead and silver carp were studied by Yan et al.^39^. They found that with *Hyriopsis cumingii* density of 0.75 ind./m^3^ and fish density of 0.075 ind./m^3^, the water quality was improved. Compared with the optimized mussel density in their research, the density in our study was higher. This was probably because the environment conditions in two systems were different, and without the competition of fish for food, more mussels were survived in our experiment.

In order to further analyze the high removal ability of pollutants in mussel/microalgae/bacteria system, digestive enzyme activities of mussels in the four different systems were tested. As shown in Table 4, there was no significant difference of amylase activity between mussel/microalgae and pure mussel system. However, the beneficial effects of *Bacillus subtilis* and *Bacillus licheniformis* on amylase activity of mussels were observed. Especially, the amylase activity was the highest in mussel/microalgae/bacteria system. With addition of *Bacillus subtilis* and *Bacillus licheniformis*, pepsin activities in the mussel/bacteria and mussel/microalgae/bacteria systems were significantly higher than that in pure mussel and mussel/microalgae systems. As such, the results suggested that *Bacillus subtilis* and *Bacillus licheniformis* enhanced the digestive capacity of mussels. The increase of digestive enzyme activity indicated improvement of digestive ability and metabolic level of the body, which was conducive to the growth and development of mussel. It was already observed that shrimp growth was improved by adding the *Bacillus subtilis*, due to stimulation of natural digestive enzyme activity of the host^40^. Moreover, the *Bacillus licheniformis* created a hostile environment for pathogen colonization, and this action of competitive exclusion resulted in a beneficial effect on the growth performance of mussels^41^. In brief, potential mechanism of wastewater treatment in the mussel/microalgae/bacteria system was that high content of NH_3_-N, TP, and COD was first used as nutrient source for the rapidly growth of *Chlorella vulgaris*, which was then filtered and consumed by *Hyriopsis cumingii*. During the treatment process, *Bacillus subtilis* and *Bacillus licheniformis* improved the digestive enzyme activities of mussels, thereby enhancing water quality in the batch experiments.

**Table 4:** Intestinal digestive enzyme activities of the mussel in different system. (U/mg prot).

Samples	Mussel	Mussel/algae	Mussel/bacteria	Mussel/algae/bacteria
Amylase activity	55.5 ± 2.8^c^	54.8 ± 1.9^c^	60.8 ± 3.8^b^	69.9 ± 4.7^a^
Pepsin activity	5.6 ± 0.8^b^	5.5 ± 0.4^b^	6.1 ± 0.9^a^	6.2 ± 0.5^a^

The different superscript letters indicated significant differences of the same enzyme activity among the different systems (P < 0.05).

Remediation potential of large-scale mussel/microalgae/bacteria culture was evaluated. It can be seen that NH_3_-N, TN, TP and COD concentration in the Kulv river was significantly decreased with the operation of treatment system. Moreover, the feeding mechanism of these mussels can result in direct increase of water clarity and light penetration of water column^42^. In this study, water transparency in Kulv river was increased from 1.0 to 1.4 m, as a result of the effective regulation of phytoplankton concentration and consequent decrease of chlorophyll *a* by filter feeding of mussels. It was also found that non-native dark false mussel populations improved water quality in a multi-impacted urban coastal lagoon, where high sewage-enriched effluents were discharged^43^. However, different from these non-native species which often invaded ecosystems and unbalanced ecological relationships, management of the native triangle sail mussel (*Hyriopsis cumingii*) in China was much more feasible.

The engineering cost of this project was estimated based on the construction scale of each individual project, the quantity of equipment required and the relevant unit price. Civil engineering and installation engineering were estimated according to construction and installation engineering quota standard of Anhui Province, and investment of similar projects already built. As shown in Table S1, the total investment of field project in this study was 2.8 million China Yuan, which mainly included the charges of construction work, breeding equipment, mussel/microalgae/bacteria cultivation, operations management, and so on. Compared with the traditional chemical method, the cost of biological treatment was not high^44,45^. Although mussel farming for water remediation was evaluated in lagoon^9^, pond^46^, and lake^47^, mussel/microalgae/bacteria farming in this study proved that it can be a cost-effectiveness of pollution control method, and good for the development of local aquaculture industry.

## Conclusions

*Hyriopsis cumingii* was one kind of aquatic animal with high economic value. Suspended solid and microalgae in water could be filtered by mussels that had the ability to purify the water body. It was necessary to master reasonable feeding density, to avoid high discharge of mussel’s excrement. The optimal breeding density of mussels was approximately 4 mussels/m^3^ in this study. Orthogonal experiment demonstrated that the optimal dose of *Bacillus subtilis*, *Bacillus licheniformis*, and *Chlorella vulgaris* were 0.5 mL, 1 mL, and 2 mL, respectively. Batch experiment demonstrated that mussel*/*microalgae/bacteria had the best treatment ability towards aquaculture wastewater. Along with the increase of microalgae biomass, element N and P and COD was reduced by *Chlorella vulgaris* through photosynthesis. *Hyriopsis cumingii* filtered the *Chlorella vulgaris* and organic debris to improve the transparency of water body. More importantly, *Bacillus subtilis* and *Bacillus licheniformis* improved the amylase and pepsin activity of mussels.

A field test was conducted in Kulv river in China. About 90 thousand mussels per hectare of water surface were cultivated which treated about 4500 m^3^ of aquaculture wastewater every day. After running for several months, the water quality was significantly improved. NH_3_-N, TN, TP and COD concentration in river water maintained around 0.3, 0.8, 0.3, and 30 mg/L respectively. This implied that after treatment Kulv river water might be used for industrial engineering and entertainment use. During the whole process, the operation cost was evaluated, and it was low. So, the present mussel/microalgae/bacteria system improved the water environment, and could promote the development of local aquaculture industry.

## Supplementary Information

Supplementary Information.

## References

[1] Cao L, Naylor R, Henriksson P, Leadbitter D, Metian M, Troell M, Zhang W (2015). China's aquaculture and the world's wild fisheries. Science.

[2] Wang Q, Cheng L, Liu J, Li Z, Xie S, De Silva SS (2015). Freshwater aquaculture in PR China: Trends and prospects. Rev. Aquac..

[3] Wu X, Wu H, Ye J (2014). Purification effects of two eco-ditch systems on Chinese soft-shelled turtle greenhouse culture wastewater pollution. Environ. Sci. Pollut. Res..

[4] Cao L, Wang W, Yang Y (2007). Environmental impact of aquaculture and countermeasures to aquaculture pollution in China. Environ. Sci. Pollut. Res. Int..

[5] Vymazal J, Kröpfelová L (2015). Multistage hybrid constructed wetland for enhanced removal of nitrogen. Ecol. Eng..

[6] Mook WT, Chakrabarti MH, Aroua MK (2012). Removal of total ammonia nitrogen (TAN), nitrate and total organic carbon (TOC) from aquaculture wastewater using electrochemical technology: A review. Desalination.

[7] Liu Q, Hu M, Wu Z (2015). Can mussels change phytoplankton community structure and enhance prawn production in semi-enclosed prawn ponds?. Aquac. Res..

[8] Taylor D, Saurel C, Nielsen P, Petersen JK (2019). Production characteristics and optimization of mitigation mussel culture. Front. Mar. Sci..

[9] Schernewski G, Friedland R, Buer AL (2019). Ecological-social-economic assessment of zebra-mussel cultivation scenarios for the Oder (Szczecin) Lagoon. J. Coast. Conserv..

[10] Timmermann K, Maar M, Bolding K (2019). Mussel production as a nutrient mitigation tool for improving marine water quality. Aquac. Environ. Interact..

[11] Lin JY, Ma KY, Bai ZY (2013). Molecular cloning and characterization of perlucin from the freshwater pearl mussel, *Hyriopsis cumingii*. Gene.

[12] He H, Liu X, Liu X (2014). Effects of cyanobacterial blooms on submerged macrophytes alleviated by the native Chinese bivalve *Hyriopsis cumingii*: A mesocosm experiment study. Ecol. Eng..

[13] Hu M, Wu F, Yuan M (2015). Antioxidant responses of triangle sail mussel *Hyriopsis cumingii* exposed to harmful algae *Microcystis aeruginosa* and hypoxia. Chemosphere.

[14] Kotta J, Futter M, Kaasik A (2020). Cleaning up seas using blue growth initiatives: Mussel farming for eutrophication control in the Baltic Sea. Sci. Total Environ..

[15] Filippelli R, Termansen M, Hasler B (2020). Cost-effectiveness of mussel farming as a water quality improvement measure: Agricultural, environmental and market drivers. Water Resour. Econ..

[16] Conroy JD, Edwards WJ, Pontius RA (2005). Soluble nitrogen and phosphorus excretion of exotic freshwater mussels (*Dreissena* spp.): Potential impacts for nutrient remineralisation in western Lake Erie. Freshw. Biol..

[17] Bellou S, Baeshen MN, Elazzazy AM (2014). Microalgal lipids biochemistry and biotechnological perspectives. Biotechnol. Adv..

[18] Beer LL, Boyd ES, Peters JW (2009). Engineering algae for biohydrogen and biofuel production. Curr. Opin. Biotech..

[19] Aslan S, Kapdan IK (2006). Batch kinetics of nitrogen and phosphorus removal from synthetic wastewater by algae. Ecol. Eng..

[20] Liu Y, Lv J, Feng J (2019). Treatment of real aquaculture wastewater from a fishery utilizing phytoremediation with microalgae. J. Chem. Technol. Biotechnol..

[21] Kesaano M, Sims RC (2014). Algal biofilm based technology for wastewater treatment. Algal Res..

[22] Xu M, Bernards M, Hu Z (2014). Algae-facilitated chemical phosphorus removal during high-density *Chlorella emersonii* cultivation in a membrane bioreactor. Bioresour. Technol..

[23] Sharma NK, Tiwari SP, Tripathi K (2011). Sustainability and cyanobacteria (blue-green algae): Facts and challenges. J. Appl. Phycol..

[24] Nagal S, Jain PC (2010). Feather degradation by strains of Bacillus isolated from decomposing feathers. Braz. J. Microbiol..

[25] Xie F, Zhu T, Zhang F (2013). Using *Bacillus amyloliquefaciens* for remediation of aquaculture water. Springerplus.

[26] Zokaeifar H, Babaei N, Saad CR (2014). Administration of *Bacillus subtilis* strains in the rearing water enhances the water quality, growth performance, immune response, and resistance against Vibrio harveyi infection in juvenile white shrimp, *Litopenaeus vannamei*. Fish Shellfish Immunol..

[27] Nakasaki K, Uehara N, Kataoka M (1996). The use of *Bacillus Licheniformis* HA1 to accelerate composting of organic wastes. Compos. Sci. Util..

[28] Rahayu S, Syah D, Suhartono MT (2012). Degradation of keratin by keratinase and disulfide reductase from *Bacillus* sp. MTS of Indonesian origin. Biocatal. Agric. Biotechnol..

[29] Kuebutornye FKA, Abarike ED, Lu Y (2019). A review on the application of *Bacillus* as probiotics in aquaculture. Fish Shellfish Immunol..

[30] Konstantinou ZI, Kombiadou K (2020). Rethinking suspended mussel-farming modelling: Combining hydrodynamic and bio-economic models to support integrated aquaculture management. Aquaculture.

[31] APHA-AWWA-WEF (1998). Standard Methods for Examination of Water and Wastewater.

[32] Chen L, Heath AG, Neves RJ (2001). Comparison of oxygen consumption in freshwater mussels (Unionidae) from different habitats during declining dissolved oxygen concentration. Hydrobiologia.

[33] Liu N, Li F, Ge F (2015). Mechanisms of ammonium assimilation by *Chlorella vulgaris* F1068: Isotope fractionation and proteomic approaches. Bioresour. Technol..

[34] Tu R, Jin W, Xi T (2015). Effect of static magnetic field on the oxygen production of *Scenedesmus obliquus* cultivated in municipal wastewater. Water Res..

[35] Wallace J, Champagne P, Hall G (2016). Multivariate statistical analysis of water chemistry conditions in three wastewater stabilization ponds with algae blooms and pH fluctuations. Water Res..

[36] Ministry of Ecology and Environment of the People’s Republic of China (2002). Environmental Quality Standard for Surface Water (GB 3838–2002).

[37] Jiang B, Xia W, Wu T (2021). The optimum proportion of hygroscopic properties of modified soil composites based on orthogonal test method. J. Clean. Prod..

[38] Su L, Zhang J, Wang C (2016). Identifying main factors of capacity fading in lithium ion cells using orthogonal design of experiments. Appl. Energ..

[39] Yan L, Zhang G, Liu Q (2009). Optimization of culturing the freshwater pearl mussels, *Hyriopsis cumingii* with filter feeding Chinese carps (bighead carp and silver carp) by orthogonal array design. Aquaculture.

[40] Zokaeifar H, Balcázar JL, Saad CR, Kamarudin MS, Sijam K, Arshad A, Nejat N (2012). Effects of bacillus subtilis on the growth performance, digestive enzymes, immune gene expression and disease resistance of white shrimp, *Litopenaeus vannamei*. Fish Shellfish Immunol..

[41] Shen WY, Fu LL, Li WF (2010). Effect of dietary supplementation with *Bacillus subtilis* on the growth, performance, immune response and antioxidant activities of the shrimp (*Litopenaeus vannamei*). Aquac. Res..

[42] Cerco CF, Noel MR (2010). Monitoring, modeling, and management impacts of bivalve filter feeders in the oligohaline and tidal fresh regions of the Chesapeake Bay system. Ecol. Model..

[43] Neves RAF, Naveira C, Miyahira IC (2020). Are invasive species always negative to aquatic ecosystem services? The role of dark false mussel for water quality improvement in a multi-impacted urban coastal lagoon. Water Res..

[44] Ahmadun F, Pendashteh A, Abdullah LC (2009). Review of technologies for oil and gas produced water treatment. J. Hazard. Mater..

[45] Li Y, Xu Z, Wu J (2020). Efficiency and mechanisms of antimony removal from wastewater using mixed cultures of iron-oxidizing bacteria and sulfate-reducing bacteria based on scrap iron. Sep. Purif. Technol..

[46] Gren IM (2019). The economic value of mussel farming for uncertain nutrient removal in the Baltic Sea. PLoS ONE.

[47] Wang L, Ma L, Sun J (2018). Effects of different aquaculture methods for introduced bivalves (*Hyriopsis cumingii*) on seston removal and phosphorus balance at the water–sediment interface. J. Freshw. Ecol..

