# Supplementary material for: Effective treatment of aquaculture wastewater with mussel/microalgae/bacteria complex ecosystem: a pilot study
Source: Sci Rep. 2022 Feb 10;12:2263. doi: 10.1038/s41598-021-04499-8 (PMC8831588; doi:10.1038/s41598-021-04499-8)
Supplement: Supplementary file 1 — Supplementary Information. [file 41598_2021_4499_MOESM1_ESM.doc]

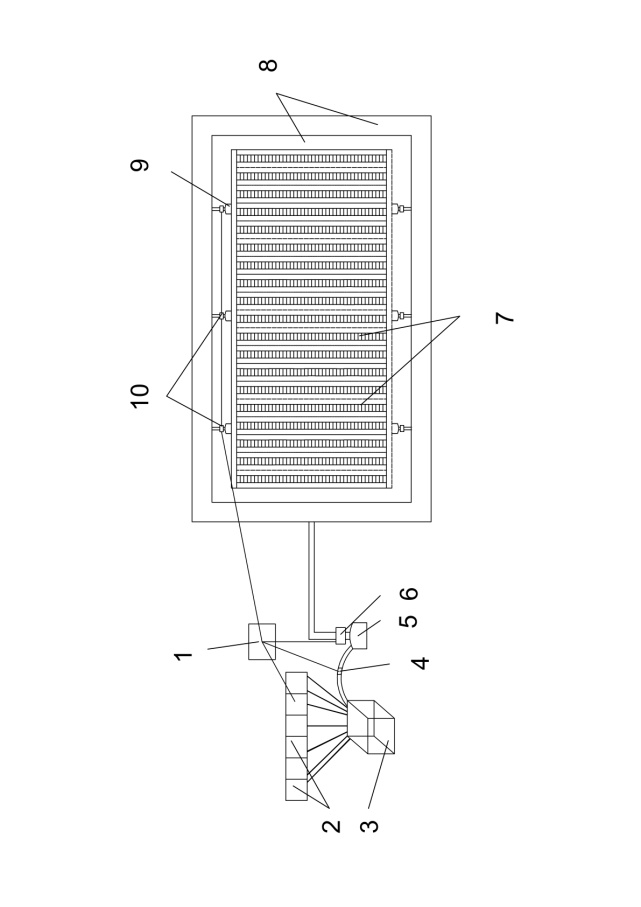


(a)


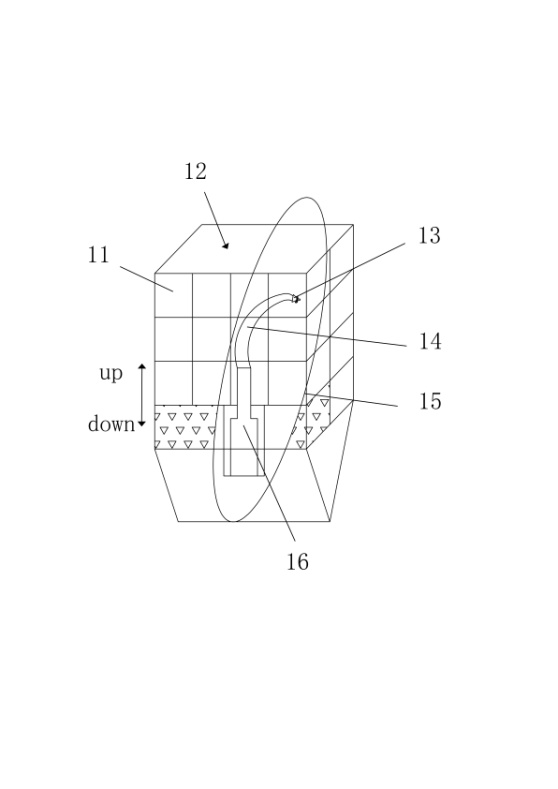


(b)

Fig.S1 (a) The automation pipe network supply feeding system for triangle sail mussel (*Hyriopsis cumingii*), 1-central controller 2-separated compounding tank 3-Integrated compounding tank 4-lifting pump 5-mixing feed station 6-feed pump 7- multiple foster boxes 8-feed delivery network 9-mixing pump feeding station 10-throttling gear; (b) The structure diagram of multiple foster boxes. 11-box body 12-opening 13-canula 14-feed tube 15- triangle sail mussel (*Hyriopsis cumingii*) 16- connecting pipe

Table S1 General expenses for wastewater treatment in field project (ten thousand yuan)

| Total expense |  |  | 280.0 |
| --- | --- | --- | --- |
| Construction work | Automatic feeding workshop, 120 square meters mobile room for staff | 3.5 |  |
|  | Light steel structure, 200 square meters greenhouse for algae cultivation | 3.0 | 8.5 |
|  | Office and management facilities (production tools, etc.) | 2.0 |  |
| Automatic  production  equipment | 113CM photosynthetic reactor | 5.0 |  |
|  | 80,000 of foster-boxes | 24.0 |  |
|  | Feed system for drip irrigation | 25.0 |  |
|  | Automatic programming control system | 9.0 | 76.0 |
|  | Line for automatic control system (50,000 meters) | 4.0 |  |
|  | Automatic control equipment (solenoid valve, valve, etc.) | 4.0 |  |
|  | Equipment of algae cultivation tank (canvas, rubber bucket, etc.) | 5.0 |  |
| Mussels and maintenance | 120,000 of pearl mussels | 128.0 |  |
|  | Transportation | 2.0 | 140.0 |
|  | Installation and hanging | 10.0 |  |
| Management | Three workers (2 years) | 45.0 | 45.0 |
| Production material | Bacteria preparation, microalgae species, medium | 15.0 | 15.0 |
| Supporting | Supporting conditions | 5.0 | 5.0 |
